# The protective effects of naproxen against interleukin-1β (IL-1β)- induced damage in human umbilical vein endothelial cells (HUVECs)

**DOI:** 10.1080/21655979.2021.1955560

**Published:** 2021-08-24

**Authors:** Yuliang Wu, Ruina Hao, Beidi Lan, Yiping Mu, Fuping Dang, Ruitao Wang

**Affiliations:** a Department of Cardiology, The First Affiliated Hospital of Xi’an Jiaotong University, Xi’an, China; b Department of Structural Heart Disease, The First Affiliated Hospital of Xi’an Jiaotong University, Xi’an, China; c Department of Medical Information Management Office, The First Affiliated Hospital of Xi’an Jiaotong University, Xi’an, China; d Department of Hepatobiliary Surgery, The First Affiliated Hospital of Xi’an Jiaotong University, Xi’an, China

**Keywords:** Naproxen, cardiovascular disease, atherosclerosis, NSAIDs, COX-2, monocyte attachment, inflammation, IL-1β

## Abstract

Non-steroidal anti-inflammatory drugs (NSAIDs) are one of the most widely used medications in the world. Naproxen is an NSAID with relatively low selectivity for cyclooxygenase-2 (COX-2), thereby having decreased risk for cardiovascular (CV) events. However, it is unclear whether naproxen might provide protection against atherosclerosis, an underlying cause of numerous cardiovascular diseases (CVDs). In the present study, we exposed human umbilical vein endothelial cells to interleukin-1β (IL-1β), a key cytokine involved in atherogenesis, with or without naproxen. Our findings indicate that naproxen could protect against IL-1β-induced damage by improving cell viability and preventing cell death. Additionally, naproxen suppressed the expression of the cytokines IL-6, IL-12, and tumor necrosis factor-α (TNF-α), and downregulated the expression of vascular endothelial growth factor (VEGF) and tissue factor (TF) induced by IL-1β. Importantly, naproxen also inhibited the attachment of monocytes to endothelial cells, which was achieved through Krüppel-like factor 6 (KLF6)-mediated reduced expression of intracellular adhesion molecule-1 (ICAM-1) and E-selectin. These findings suggest that naproxen may aid in the prevention of atherosclerosis by exerting cardioprotective effects beyond low COX-2-selectivity.

## Graphical Abstract

**Figure:**
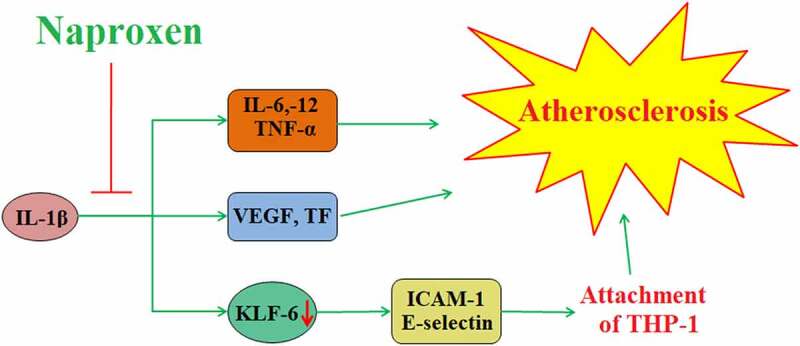

## 1. Introduction

Cardiovascular disease (CVD) refers to a broad range of diseases involving the cardiovascular (CV) system, including coronary artery disease, hypertension, stroke, and ischemic heart disease, among others. According to reports, 17.9 million people died of cardiovascular disease in 2018, and this number will increase to 23.8 million by 2030 [1]. CVD has become a serious society and economic burden around the world. Atherosclerosis (AS) plays a major role in the development and progression of these and other CVDs. Characterized by the accumulation of fatty plaques on the arterial wall, AS is a complex disease involving numerous cell types and signaling mechanisms [2]. Endothelial cells (ECs) line the luminal surface of vessel walls and perform the important functions of regulating vascular tone and angiogenesis, reacting to mechanical and immunological stimuli, and transporting lipids and other solutes across the endothelial barrier, among other things. Typically, ECs promote the free flow of blood by discouraging the attachment of immune cells to the endothelium. In atherosclerotic conditions, however, ECs become activated and lose their normal protective functionality, instead taking on a pro-inflammatory and pro-atherosclerotic phenotype. These dysfunctional ECs secrete proinflammatory cytokines, such as interleukins (ILs) and tumor necrosis factor-α (TNF-α), as well as adhesion molecules, such as intracellular adhesion molecule-1 (ICAM-1) and E-selectin. The expression of these molecules drives the recruitment of immune cells to the endothelium, where they adhere to the vessel wall and infiltrate the intimal space. The accumulation of lipids and leukocytes within the intimal space leads to the formation of foam cells and fatty plaques, which are the hallmarks of AS [3,4].

Inflammation plays a central role in the initiation and progression of AS. Interleukin-1β (IL-1β) is one of the main forms of circulating IL-1β. In normal physiology, IL-1β exists in its inactive form pro-IL-1β, but at the onset of the inflammatory response, pro-IL-1β becomes activated through the inflammasome, thereby driving systemic inflammation [5,6]. Activated IL-1β triggers ECs to drive the recruitment and transmigration of immune cells by producing cytokines, chemokines, and cellular adhesion molecules [7]. IL-6 and IL-12 are two such cytokines that have been implicated in the pathogenesis of AS. Studies have shown that increased serum levels of IL-6 and IL-12 are positively correlated with the occurrence and severity of AS [8,9]. Inhibition of the expression of pro-inflammatory cytokines caused by IL-1β is considered as a potential treatment and prevention strategy for AS, but the mechanisms involved still need to be elucidated [10].

Non-steroidal anti-inflammatory drugs (NSAIDs) are one of the safest and most commonly used over-the-counter medications in the world. The analgesic, antipyretic, and anti-inflammatory properties of NSAIDs make their use applicable in a wide range of situations. NSAIDs work by inhibiting the activity of cyclooxygenase-1 (COX-1) and COX-2, also known as prostaglandin-endoperoxide synthase (PGHS), which are involved in prostaglandin/prostanoid production, hemostasis, and CV function. While both COX-1 and COX-2 are involved in prostaglandin production, COX-2 is the primary producer of prostacyclin (PGI_2_), which has been shown to exert cardioprotective effects [11]. The use of NSAIDs, and especially those with higher affinity for COX-2, has been associated with increased risk of CV events, including myocardial infarction, stroke, and heart failure. Owing to its relatively low selectivity for COX-2, naproxen is considered to have a better CV risk profile compared to other NSAIDs. Naproxen is another NSAID with low affinity for COX-2, and is well-recognized for its cardioprotective effects at low doses [12].

Based on these findings reported in previous studies, we recognized that treatments which prevent inflammatory response and monocyte attachment should be therapeutic strategies for AS. However, whether naproxen exerts potential benefits in the development of AS is still unknown. Thus, in the present study, we investigated the impact of naproxen on endothelial cells stimulated with the pro-atherogenic inflammatory cytokine IL-1β to determine whether naproxen might offer endothelial protective effects on inflammatory response and monocyte attachment.

## 2. Materials and methods

### 2.1. Cell culture and treatment

Human umbilical vein endothelial cells (HUVECs) and THP-1 monocytes were purchased from the American Type Culture Collection (ATCC). HUVECs were cultured in EGM-2 bullet kit medium with 10% fetal bovine serum (FBS) and 0.1% penicillin/streptomycin. For the treatment experiments, cells were challenged with 10 ng/mL IL-1β in the presence or absence of 5 and 10 µM naproxen.

### 2.2. CCK-8 assay

HUVECs were seeded into 96-well plates at a density of 3 × 10^3^ and a final volume of 100 μL per well. After the indicated treatment, 10 µL Cell Counting Kit‐8 (CCK‐8) solution was added to each well, followed by an additional 4 hours of culturing at 37°C. Absorbance was measured at 450 nm using a Multiskan FC microplate reader (Thermo Fisher Scientific) to index cell viability.

### 2.3. LDH release

The release of lactate dehydrogenase (LDH) from cells serves as an indicator of cell death [13]. A commercial LDH kit (#ab65391, Abcam, USA) was used to determine the release of LDH from treated HUVECs. Briefly, 50 μL of the culture medium was collected from each well. The collected medium was then mixed with an equal amount of reaction buffer and incubated at room temperature for 30 min in darkness. A fluorescent microscope was used to measure the OD value at 490 nm.

### 2.4. Real-time PCR

The mRNA expression levels of IL-6, IL-12, TNF-α, vascular endothelial growth factor (VEGF), tissue factor (TF), and Krüppel-like factor 6 (KLF6) were measured using real-time polymerase chain reaction (PCR) analysis. Briefly, the total RNA was isolated from treated HUVECs and purified using a High Pure RNA kit (Roche). Then, reverse transcription PCR was performed using 1 µg purified RNA from each treatment group to generate cDNA using iScript SuperMix (Invitrogen, USA). The generated cDNA was used for real-time PCR analysis using the SYBER Green method (Roche) in a 20 µL reaction system.

### 2.5. Western blot analysis

Briefly, cells from the different treatment groups were separated using polyacrylamide gel electrophoresis (PAGE) and then transferred onto polyvinylidene fluoride (PVDF) membranes. The membranes were then incubated with the corresponding primary and secondary antibodies, and an enhanced chemiluminescence kit (Sigma-Aldrich, USA) was used to visualize the immunoblots [14].

### 2.6. ELISA

To determine the protein secretion levels of IL-6, IL-12, TNF-α, VEGF, TF, ICAM-1, and E-selectin, commercial ELISA kits were purchased from R&D Systems. Briefly, the cell culture medium was collected and transferred into 96-well plates, followed by incubation overnight at 4 °C. Then, the samples were sequentially incubated with detection antibody, secondary antibody, and stabilized chromogen. The reaction was stopped using stop solution. The secretions of the target genes were indexed by measuring the OD value at 450 nm.

### 2.7. Calcein-AM staining

For our cellular adhesion experiments, THP-1 cells were labeled with 5 µM of the green-fluorescent cell-permanent dye calcein-AM (Thermo Fisher Scientific, USA) by incubating at 37 °C for 30 minutes. Then, THP-1 monocytes at a density of 5 × 10^5^ were incubated with 1 × 10^5^ confluent HUVECs in DMEM at 37 °C for 2 h. The non-adherent cells were washed away, and then the number of THP-1 cells adhered to HUVECs was visualized and quantified using a fluorescence microscope with excitation at 485 nm and emission wavelengths at 530 nm.

### 2.8. Statistical analysis

Results are expressed as means ± standard deviation (S.D.). The significance of differences between the groups was assessed using SPSS statistical software. Statistical significance was determined based on analysis of variance (ANOVA), followed by Bonferroni’s post-hoc test. P values of less than 0.05 were considered to be statistically significant.

## 3. Results

In order to examine the potential benefits of naproxen in AS, we used HUVECs stimulated with IL-1β to establish an *in vitro* cell model. We investigated the effects of naproxen on the expression of proinflammatory cytokines, VEGF, TF, endothelial adhesion molecules, as well as the attachment of monocytes. We found that naproxen had a robust beneficial property against IL-1β- induced insults in HUVECs. Notably, we proved that these effects are mediated by KLF6.

### 3.1. Effects of naproxen on cell viability and release of LDH

**Figure 1.**
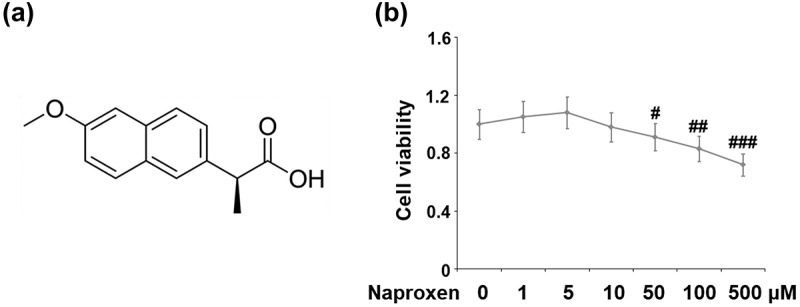
Cytotoxicity of naproxen in HUVECs. Cells were stimulated with naproxen at the concentrations of 1, 5, 10, 50, 100, and 500 μM for 24 h. (a). Molecular structure of naproxen; (b). Cell viability of HUVECs was measured using CCK-8 kit (#, ##, ###, P < 0.05, 0.01, 0.005 vs. vehicle group)

The molecular structure of naproxen is shown in Figure 1a. We began by testing the effects of various doses of naproxen ranging from 1 to 500 µM on the cell viability of HUVECs. A CCK-8 kit was used to detect cell viability. The results in Figure 1b show that cell viability was not significantly affected when the concentration of naproxen was lower than 10 µM. However, treatment with 50, 100, and 500 µM further reduced cell viability in a dose-dependent manner. These results suggested that when the concentration of naproxen was higher than 10 µM, it had a negative impact on cell viability. Therefore, we chose to use doses of 5 and 10 µM for the following IL-1β stimulation experiments.

**Figure 2.**
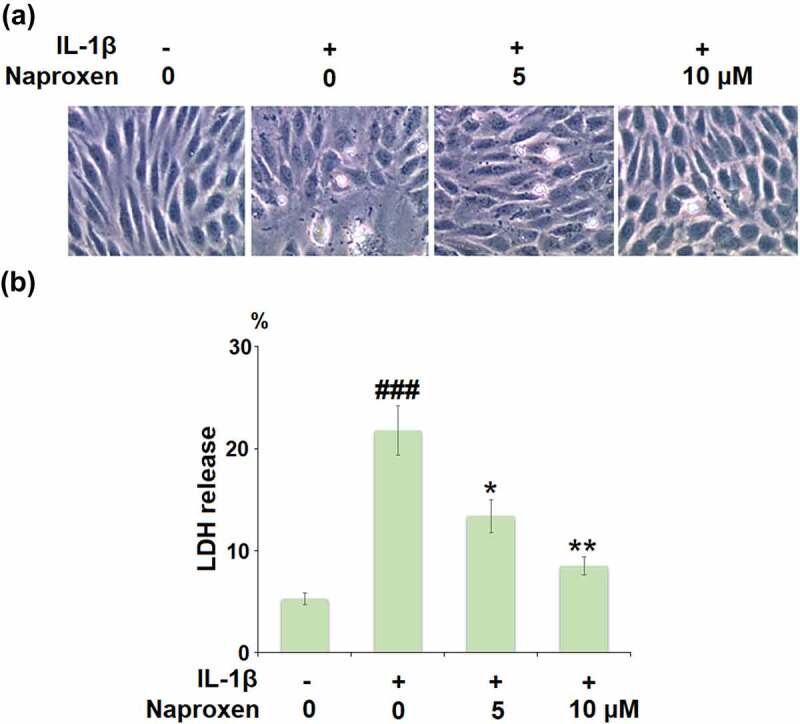
Naproxen prevents IL-1β-induced LDH release in HUVECs. Cells were incubated with IL-1β (10 ng/mL) with or without naproxen (5, 10 μM) for 24 h. (a). Morphology of HUVECs in different culture conditions; (b). LDH release was measured using a commercial kit (###, P < 0.005 vs. vehicle group; *, **, P < 0.05, 0.01 vs. IL-1β group)

The morphology of HUVECs in the different culture conditions is shown in Figure 2a. Measuring the release of LDH is a common method for quantifying irreversible cell death due to membrane rupture. As shown in Figure 2b, stimulation with IL-1β drastically increased the release of LDH from HUVECs from 5.3% to 21.6%, while cells treated with 5 and 10 µM naproxen demonstrated a significant inhibitory effect against IL-1β-induced release of LDH, and reduced LDH release to 13.4% and 8.5%, respectively, thereby indicating a protective effect of naproxen against IL-1β-induced cell death.

### 3.2. Naproxen suppresses IL-1β-induced expression of proinflammatory cytokines

**Figure 3.**
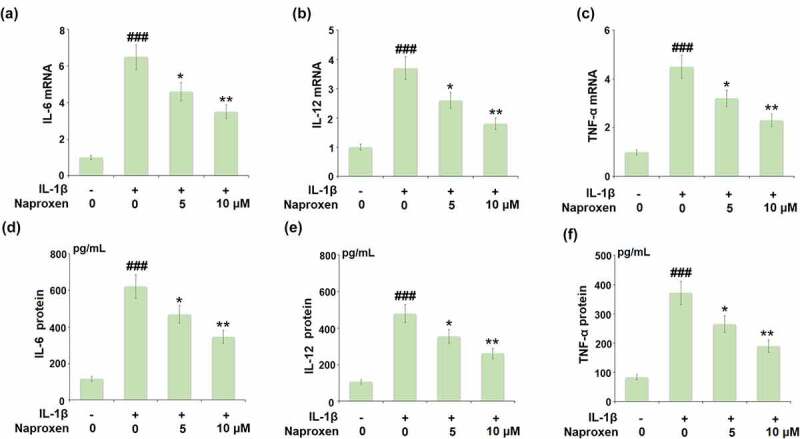
Naproxen suppresses IL-1β-induced expression of pro-inflammatory cytokines in HUVECs. Cells were incubated with IL-1β (10 ng/mL) with or without naproxen (5, 10 μM) for 24 h. (a). mRNA of IL-6 (1, 6.5, 4.6, 3.5); (b). mRNA of IL-12; (c). mRNA of TNF-α; (d). Secretions of IL-6; (e). Secretions of IL-12; (f). Secretions of TNF-α (###, P < 0.005 vs. vehicle group; *, **, P < 0.05, 0.01 vs. IL-1β group)

The expression of IL-1β as well as the expression of other proinflammatory cytokines triggered by IL-1β are significant events in the initiation and progression of atherosclerotic CVD. Thus, we employed real-time PCR and ELISA analyses to determine the effects of naproxen on the mRNA and protein expression of pro-inflammatory cytokines induced by IL-1β in HUVECs. As shown in Figure 3a-3c, naproxen displayed a significant inhibitory effect against IL-1β-induced expression of IL-6, IL-12, and TNF-α at the mRNA level. Similarly, the results in Figure 3d-3f show that IL-1β increased the secretions of IL-6, IL-12, and TNF-α from 116.8, 105.3, and 83.6 pg/mL to 621.7, 479.2, and 371.7 pg/mL, however, the two doses of naproxen dose-dependently reduced them to 468.9, 353.8, 264.3 pg/mL, as well as 345.3, 261.5, and 188.9 pg/mL.

### 3.3. Naproxen inhibits IL-1β-induced expression of VEGF and TF

**Figure 4.**
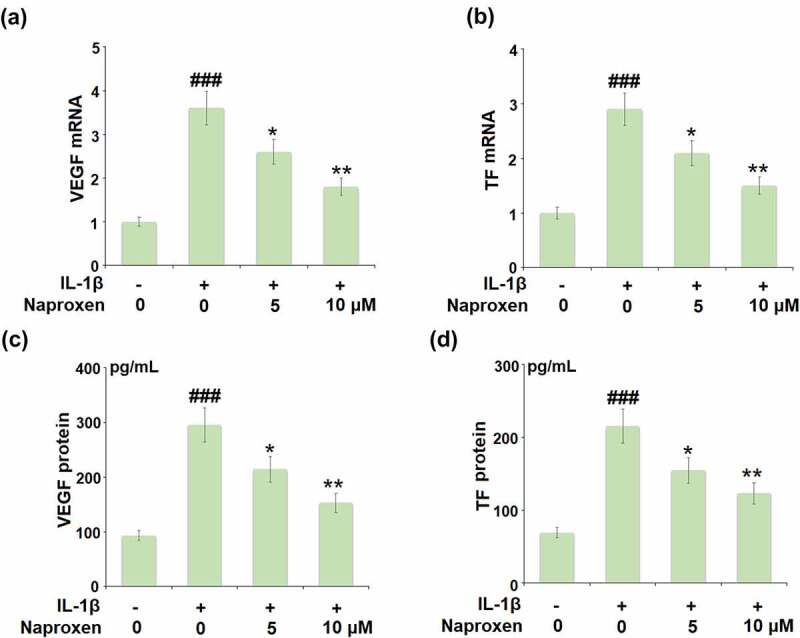
Naproxen inhibits IL-1β-induced expression of VEGF and tissue factor (TF) in HUVECs. Cells were incubated with IL-1β (10 ng/mL) with or without naproxen (5, 10 μM) for 24 h. (a). mRNA of VEGF; (b). mRNA of TF; (c). Production of VEGF; (d). Production of TF (###, P < 0.005 vs. vehicle group; *, **, P < 0.05, 0.01 vs. IL-1β group)

Increased expression of VEGF and TF is associated with the development of atherosclerosis [15,16]. The results in Figure 4a-4b reveal that while stimulation with IL-1β roughly tripled the mRNA expression of VEGF and TF, this action was suppressed by the two doses of naproxen, with the higher dose reducing the increase by about half. The results of ELISA in Figure 4c-4d demonstrate a similar inhibitory effect of naproxen at the protein level.

### 3.4. Naproxen inhibits the attachment of monocytes to endothelial cells

**Figure 5.**
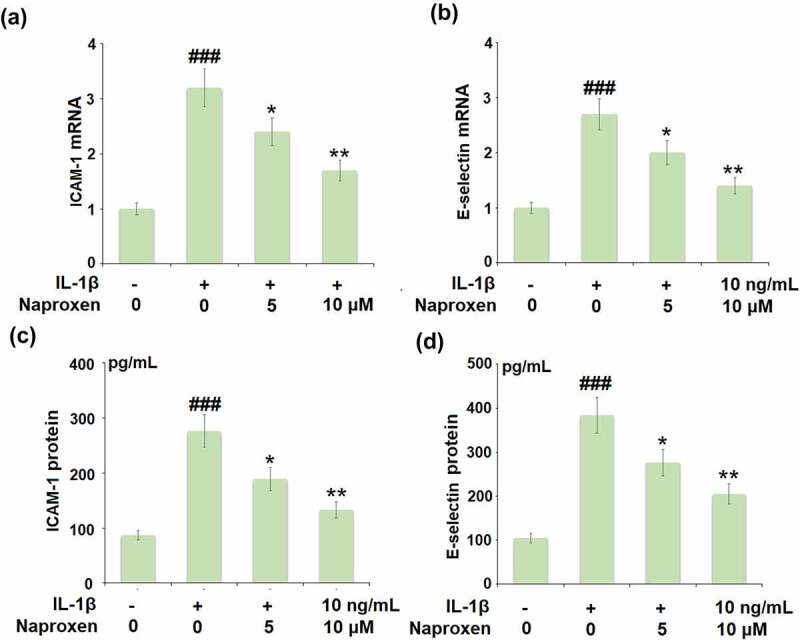
Naproxen reduces IL-1β-induced expression of ICAM-1 and E-selectin. Cells were incubated with IL-1β (10 ng/mL) with or without naproxen (5, 10 μM) for 24 h. (a). mRNA of ICAM-1; (b). mRNA of E-selectin; (c). Production of ICAM-1; (d). Production of E-selectin (###, P < 0.005 vs. vehicle group; *, **, P < 0.05, 0.01 vs. IL-1β group)

**Figure 6.**
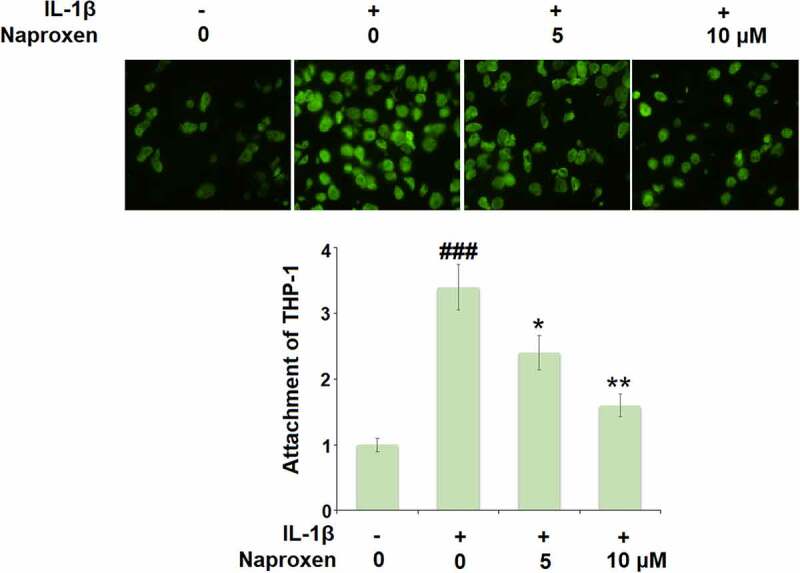
Naproxen prevents IL-1β-induced attachment of THP-1 monocytes to HUVECs. Cells were incubated with IL-1β (10 ng/mL) with or without naproxen (5, 10 μM) for 24 h. Attachment of THP-1 monocytes to HUVECs was measured using Calcein-AM staining (###, P < 0.005 vs. vehicle group; *, **, P < 0.05, 0.01 vs. IL-1β group)

To investigate the effects of naproxen on monocyte-endothelial cell adhesion, we first measured the expression levels of the cellular adhesion molecules ICAM-1 and E-selectin. As shown in Figure 5a-5d, stimulation of HUVECs with IL-1β increased the expression of both ICAM-1 and E-selectin at the mRNA and protein levels by roughly 3-fold. However, treatment with naproxen significantly reduced the increasing expression of ICAM-1 and E-selectin induced by IL-1β. Moreover, 10 µM naproxen inhibited the expression of ICAM-1 and E-selectin to approximately half. Next, we performed a cellular adhesion assay using THP-1 monocytes to determine whether the inhibitory effect of naproxen on ICAM-1 and E-selectin could translate to reduced attachment of monocytes to HUVECs. As shown in Figure 6, the results of Calcein-AM staining revealed that exposure to IL-1β more than tripled the number of adhered monocytes, but the two doses of naproxen counteracted this effect, with the higher dose reducing the number of attached monocytes by about half, which is consistent with the inhibitory effect of naproxen on the expression of cellular adhesion molecules ICAM-1 and E-selectin.

### 3.5. The anti-cellular adhesion effect of naproxen is mediated through KLF6

**Figure 7.**
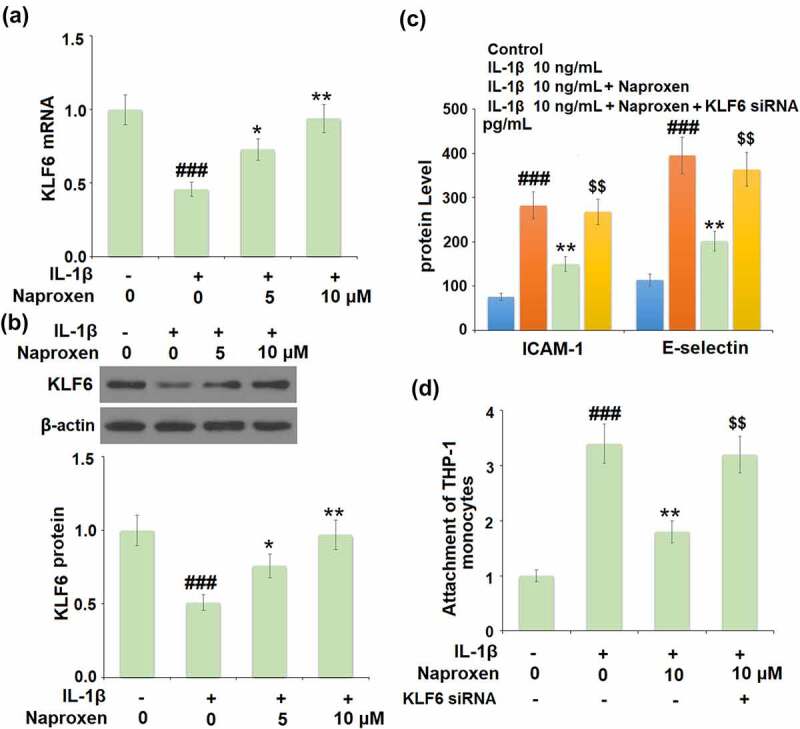
The protective effects of naproxen against IL-1β-induced attachment of THP-1 monocytes to HUVECs are dependent on KLF6. (a-b). Cells were incubated with IL-1β (10 ng/mL) with or without naproxen (5, 10 μM) for 24 h. mRNA and protein levels of KLF6 were measured; (c-d). Cells were transfected with KLF6 siRNA, followed by stimulation with IL-1β (10 ng/mL) with or without naproxen (10 μM) for 24 h. Production of ICAM-1 and E-selectin was measured by ELISA. Attachment of THP-1 monocytes to HUVECs was measured using Calcein-AM staining (###, P < 0.005 vs. vehicle group; **, P < 0.01 vs. IL-1β group; $$, P < 0.01 vs. IL-1β+ IL-1β group)

Finally, we investigated the role of the KLF6 signaling pathway in mediating the anti-cellular adhesion effect of naproxen in IL-1β-challenged HUVECs. Firstly, we determined whether the expression level of KLF6 changes in response to IL-1β in cells treated with naproxen. As shown in Figure 7a-7b, treatment with IL-1β reduced the mRNA and protein expression levels of KLF6 by roughly half. However, the addition of 5 and 10 µM naproxen significantly suppressed this inhibitory effect in a dose-dependent manner. Next, we blocked the activity of KLF6 using siRNA to explore whether the anti-cellular adhesion effect of naproxen is dependent on KLF6 signaling. As shown in Figure 7c-7d, blockade of KLF6 abolished the inhibitory effect of naproxen against IL-1β-induced increased ICAM-1 and E-selectin expression, respectively. The results of Calcein-AM staining confirmed that blockade of KLF6 also abolished the ability of naproxen to prevent the attachment of monocytes to endothelial cells.

## 4. Discussion

NSAIDs are among the most widely used pain medications in the world, but have been associated with increased CV risk. The use of NSAIDs is especially high among elderly patients suffering from arthritic musculoskeletal pain, and has been shown to contribute to the greater incidence of CVD-related death and all-cause mortality in patients with active osteoarthritis [17,18]. The mechanism of action of NSAIDs involves selective or nonselective inhibition of COX-1 and/or COX-2. COX-1 and COX-2 are enzymes involved in the synthesis of inflammatory prostaglandins, which also confer certain CV and gastrointestinal protective effects. While COX-1 inhibition was previously regarded as a source of gastrointestinal complications, more recent research has suggested otherwise [19]. Nonselective NSAIDs, such as ibuprofen, diclofenac, aspirin, and naproxen, have varying degrees of selectivity for COX-1/COX-2. Of these, aspirin has the highest selectivity for COX-1 at 150-fold [20] followed by naproxen, with 5-fold selectivity for COX-1 [12]. Meanwhile, COX-2 selective NSAIDs, such as celecoxib, etorocoxib, and lumiracoxib, have been shown to carry more than 30% greater risk of myocardial infarction compared to placebo [17]. Numerous studies have agreed that naproxen is a safer choice of NSAID in regards to CV risk [21], but it remains unclear whether the properties of naproxen can translate to CV protection, particularly in terms of AS. Therefore, it’ s meaningful to examine whether naproxen possesses a protective effect against IL-1β- induced inflammatory injuries in endothelial cells.

AS is a complex disease characterized by the accumulation of lipid-laden foam cells within the arterial intimal space, which culminates in the formation of fatty plaques in large and medium arteries. Left untreated, AS can lead to ischemic heart disease, myocardial infarction, or stroke due to arterial occlusion or plaque rupture [22]. Previous research has highlighted the roles of both COX-1 and COX-2 in modulating atherosclerotic plaque stability [23,24]. However, there has been limited research on the specific effects of the nonselective COX inhibitor naproxen in atherogenesis. The anti-inflammatory abilities of naproxen have been thoroughly documented throughout the past decades [25–27]. In the present study, we found that naproxen could reduce the expression of the proinflammatory cytokines IL-6, IL-12, and TNF-α induced by IL-1β, one of the primary cytokines involved in athreogenesis. IL-6 has been shown to be an independent predictor of future vascular events, and inhibition of IL-6 was shown to reduce the risk of incident cardiovascular events, cardiovascular death, and all-cause mortality in patients taking canakinumab [28]. The IL-12 family of cytokines consists of IL-12, IL-23, IL-27, and IL-35. The normal expression of these family members has been shown to be disrupted in CVDs including AS, coronary artery disease, atrial fibrillation, viral myocarditis, and others [29]. TNF-α is another well-known cytokine that has been widely studied for its pro-atherosclerotic role [30,31]. Consistent with our results, previous research has shown that treatment with naproxen at doses of 5–20 µM could reduce the expression of TNF-α in a rat model of Alzheimer’s disease [32]. These findings suggest that the anti-inflammatory effects of naproxen may be applicable in the context of AS due to its influence on key AS-associated inflammatory cytokines.

The formation of new blood vessels within atherosclerotic plaques is a major event in AS plaque formation and stability. VEGF contributes to atherogenesis by increasing vascular permeability, promoting EC proliferation and migration, triggering monocyte chemotaxis, and regulating thrombogenesis [33]. VEGF is also involved in plaque formation and instability. Although the administration of anti-VEGF drugs has resulted in adverse CV events in some patients, more recent research suggests that VEGF inhibition within plaques may prevent plaque rupture by suppressing intraplaque hemorrhaging and angiogenesis [34]. TF is most widely recognized as a key player in the process of coagulation. In AS, inflammatory factors initiate the expression of TF by vascular smooth muscle cells, monocytes, and extracellular vesicles, which leads to FXa and thrombin expression in response to TF-mediated coagulation [35]. Here, we found that treatment with naproxen inhibited the expression of VEGF and TF induced by IL-1β in HUVECs, thereby suggesting potential protective effects against angiogenesis, plaque formation, and plaque rupture.

The adhesion of monocytes to endothelial cells is a critical event in the development and progression of AS. In the present study, we found that treatment with naproxen suppressed monocyte adhesion to ECs by inhibiting the expression of ICAM-1 and E-selectin. Importantly, this effect was found to be dependent on KLF6 activation. The role of adhesion molecules in AS has been thoroughly studied [36–38], but the interaction between EC-expressed adhesion molecules and naproxen is not clearly understood. Upregulation of COX-2 has been shown to increase ICAM-1 expression in brain ECs [39]. In the present study, naproxen inhibited the expression of ICAM-1, but it is unclear whether COX-2 inhibition was involved. On the other hand, we determined that the anti-cellular adhesion effect of naproxen was dependent on KLF6 signaling. KLF6 is a zinc finger transcription factor that has been shown to regulate the processes of vascular remodeling and angiogenesis. Previous research has identified upregulation of KLF6 could inhibit the attachment of monocytes to endothelial cells by reducing the expression of monocyte chemoattractant protein-1 (MCP-1) and vascular endothelial adhesion molecule-1 (VCAM-1) [40]. This complements our finding that naproxen could inhibit monocyte attachment via KLF6-mediated reduced expression of ICAM-1 and E-selectin. Conversely, a recent study suggested that KLF6 may contribute to macrophage activation and atherogenesis [41]. Further research into the role of KLF6 in atherosclerosis and the range of potential for naproxen as an anti-AS treatment is still necessary. In our future study, we might use the KLF6 deficient endothelial cells or KLF6 deficient mice to confirm the physiological function of KLF2 in mediating the protective effects of naproxen.

## Conclusion

In conclusion, our results provide evidence that naproxen may exert atheroprotective effects by reducing inflammation and atherogenesis and inhibiting the adhesion of monocytes to ECs via modulating the KLF6 signaling pathway. In the future, we plan to expand our study of the role of naproxen in AS using *in vitro* experimentation with different cell types as well as *in vivo* studies with animal models.
